# Impact of COVID-19 on gynecologic and obstetrical services at two large health systems

**DOI:** 10.1371/journal.pone.0269852

**Published:** 2022-06-16

**Authors:** Angela L. Liang, Lindsay C. Turner, Kristen M. Voegtline, Sarah B. Olson, Brian Wildey, Victoria L. Handa

**Affiliations:** 1 Johns Hopkins University School of Medicine, Baltimore, Maryland, United States of America; 2 Department of Obstetrics and Gynecology, Division of Urogynecology, Allegheny Health Network, Pittsburgh, Pennsylvania, United States of America; 3 Biostatistics, Epidemiology and Data Management (BEAD) Core, Johns Hopkins University School of Medicine, Baltimore, Maryland, United States of America; 4 Department of Gynecology and Obstetrics, Johns Hopkins University School of Medicine, Baltimore, Maryland, United States of America; Dipartimento di Scienze Mediche e Chirugiche (DIMEC), Orsola Hospital, ITALY

## Abstract

**Background:**

The COVID-19 pandemic disrupted medical care in the US, leading to a significant drop in utilization of some types of health services. We sought to quantify how the pandemic influenced obstetrics and gynecology care at two large health care organizations.

**Materials and methods:**

Comparing 2020 to 2019, we quantified changes to obstetrics and gynecology care at two large health care organizations in the United States, Allegheny Health Network (in western Pennsylvania) and Johns Hopkins University (in Maryland). The analysis considered the numbers of surgical encounters, in-person visits, and telemedicine visits. For each system, we quantified temporal changes in surgical volume, in-person and telemedicine visits, and financial impact related to professional fee revenues. We used segmented regression to evaluate longitudinal effects.

**Results:**

At both institutions, the volume of care was similar in the first few months of 2020 compared to 2019 but dropped precipitously in March 2020. From April to June 2020, surgical volumes were 67% of the same period in 2019 at Allegheny Health and 48% of the same period in 2019 at Johns Hopkins. During that same interval, televisits accounted for approximately 21% of all ambulatory care at both institutions. Although surgical and ambulatory volumes recovered in the second half of 2020, annual surgical volumes in 2020 were significantly lower than 2019 at both institutions (p<0.05) and 2020 ambulatory volumes remained significantly lower at Johns Hopkins (*p* = .0006). Overall, revenues in 2020 were 91% of 2019 revenues for both institutions.

**Conclusions:**

Obstetrical and gynecologic ambulatory visits and gynecologic surgeries were sharply reduced during the COVID-19 pandemic. Although care volumes returned to 2019 levels in late 2020, we observed an overall reduction in the volume of care provided and a 9% reduction in professional revenue for both institutions.

## Introduction

The first cases of COVID-19 in the United States were reported in January 2020 [1]. Soon after, the federal and state governments declared a state of emergency [2–4]. Stay-at-home orders went into effect, non-essential businesses suspended in-person activities, and universal masking became required [5, 6]. Healthcare organizations similarly adapted. Elective procedures were cancelled, hospital visitors were restricted, and guidelines regarding telehealth were relaxed [7–9]. Collectively, these measures were intended to mitigate the spread of COVID-19.

Early in the pandemic, ambulatory visits at some medical centers in the U.S. decreased substantially, with a simultaneous increase in telehealth visits [10, 11]. However, conversion of in-person care to telemedicine care was variable across specialties [12]. Surgery volumes were also reduced across multiple specialties [13–16]. Surprisingly, even volumes for some types of emergency department care were attenuated [17], including for some gynecologic conditions [18]. The impact of the COVID-19 pandemic on obstetrical and gynecological ambulatory care is unknown. As obstetrical and gynecological services include some discretionary or elective care (such as preventative care) and some types of care that cannot be deferred (such as childbirth), we sought to investigate how the pandemic influenced the provision of both surgical and ambulatory care across our specialty.

The purpose of this study was to quantify the impact of COVID-19 on the volume of gynecological and obstetrical care provided at two large health care organizations: Allegheny Health Network in western Pennsylvania and Johns Hopkins University in Maryland. First, we sought to describe temporal changes in the volume of gynecologic surgical care provided during this period. Second, we examined temporal changes to the volume of in-person and telemedicine ambulatory visits. The third objective was to quantify the financial impact to professional fee revenues.

## Materials and methods

The research was conducted under a cooperative agreement between Johns Hopkins Medicine and the Allegheny Health Institute. The Johns Hopkins Medicine Institutional Review Boards evaluated this research and designated this study as exempt from formal review (#IRB00260775); the Allegheny Health Institute ceded authority for human subjects’ review to the Johns Hopkins Medicine via a Master Common Reciprocal Institutional Review Board Authorization Agreement.

Data for this study were extracted from the electronic medical record and related business intelligence software. The data were obtained as a “limited data set”, including identifiable patient information as defined in the Privacy Regulations issued under the Health Insurance Portability and Accountability Act (HIPAA). As such, informed consent was not obtained from individual patients. For all analyses, we considered the data for each of the two health systems (Allegheny Health and Johns Hopkins) separately. All analyses were performed with SAS 9.4 and statistical significance was defined as 0.05.

Our first aim was to describe and quantify the changes in gynecologic surgical care provided during the pandemic. To address this aim, we identified all surgical procedures performed by all gynecologic surgeons at each health system using Current Procedural Terminology (CPT) codes to identify relevant procedures (see Supplement). We calculated the number of surgery encounters per week for each week of 2019 and 2020. If a patient underwent more than one surgical procedure on a specific date, we considered that as a single surgical encounter. We excluded the first and last week of each year because these weeks represented partial weeks based on date range (and therefore underestimated the true count). For the remaining 50 weeks, we calculated the ratio of surgical encounters in 2020 to the corresponding week in 2019 (separately for each of our two health systems). These ratios were plotted as a function of time. We did not consider changes to obstetrical delivery volumes or surgical procedures related to delivery, as we hypothesized these volumes would be more directly influenced by long-term trends in birth rates, rather than by temporal changes related to COVID-19.

We used a similar approach to compute weekly ambulatory visit volumes, including both in-person and telemedicine visits. For in-person visits, we first determined the total number of completed ambulatory obstetrical and gynecological visits for each week of 2019 and 2020. We also determined visit volumes for specific subsets of care, including prenatal care, gynecologic annual preventative care, and gynecologic problem-focused care. Ambulatory visit categories were classified based on how each appointment was scheduled, rather than by CPT or International Statistical Classification of Disease (ICD) coding associated with the visit.

In a separate analysis, we calculated the proportion of televisits as a function of all ambulatory visits for each week of 2020. Televisits in 2019 were not considered, due to low or zero frequencies prior to 2020. We also calculated the proportion of televisits for each subspecialty of obstetrics and gynecology. The following specialties were included: family planning, female pelvic medicine and reconstructive surgery, gynecologic oncology, maternal fetal medicine, and reproductive endocrinology and infertility. Family planning visits were identified by location (e.g., for both institutions, these visits occurred in designated subspecialty clinics). For all other subspecialties, visits were attributed to that subspecialty if they were completed with a board-certified subspecialist or with an advanced practice provider associated with that subspecialty practice.

To evaluate the impact of the COVID-19 pandemic over time for each outcome, we employed an interrupted time series analytic approach that utilizes the statistical model of segmented regression to evaluate longitudinal effects. Segments in the time series were defined by key change points occurring in 2020 (i.e., discrete phases of the pandemic), guided by established pandemic impacts on national health care delivery as well as empirical data specific to the two health systems in the current study. Prior to evaluation of discontinuities in encounter volumes over time within the segmented regression model, and given the repeated measures in our data, we evaluated autocorrelation (e.g., each week will be more similar to adjacent weeks than to temporally distant weeks) and seasonality (e.g., typical calendar-related variations in care). We did not find evidence of either within Allegheny Health (autocorrelation: Durbin-Watson *p range* = .50 -.89; seasonality: Dickey-Fuller Unit Root Test *p =* .001) or Johns Hopkins (autocorrelation: Durbin-Watson *p range =* .33 - .41; seasonality: Dickey-Fuller Unit Root Test *p =* .001).Therefore, we proceeded with a standard segmented regression model that yielded tests of level and slope change across segments.

Our final aim was to describe professional fee revenue for each institution, by month, for 2019 and 2020. We calculated the ratio of revenues for each month in 2020, compared to the corresponding month in 2019. We report monthly data (rather than weekly data) for this analysis because administrative practices may influence week-to-week collections. Also, as there may be a significant lag between the delivery of care and revenue received, we anticipated that the change points for revenue might differ from the change points identified and applied for surgical or ambulatory volumes.

## Results

Segments in the time series were defined by three key change points occurring in 2020 (i.e., discrete phases of the pandemic) [1–9]. The first change point occurred during the week of March 8–15, 2020 when local and state restrictions were enacted in response to the pandemic onset [1–3]. The second change point, the week of April 5–11, 2020, was the point of maximum impact across the two health systems. Finally, the third change point, the week of June 28-July 4, 2020, was the point at which restrictions regarding elective surgical procedures were relaxed at both institutions. Using these three key change points, 2020 was segmented into four time periods for analysis: period 1 (pre-pandemic) as prior to March 8, 2020; period 2 as March 8 to April 4, 2020; period 3 as April 5 to June 27, 2020; and period 4 as June 28 to December 26, 2020.

**Fig 1:**
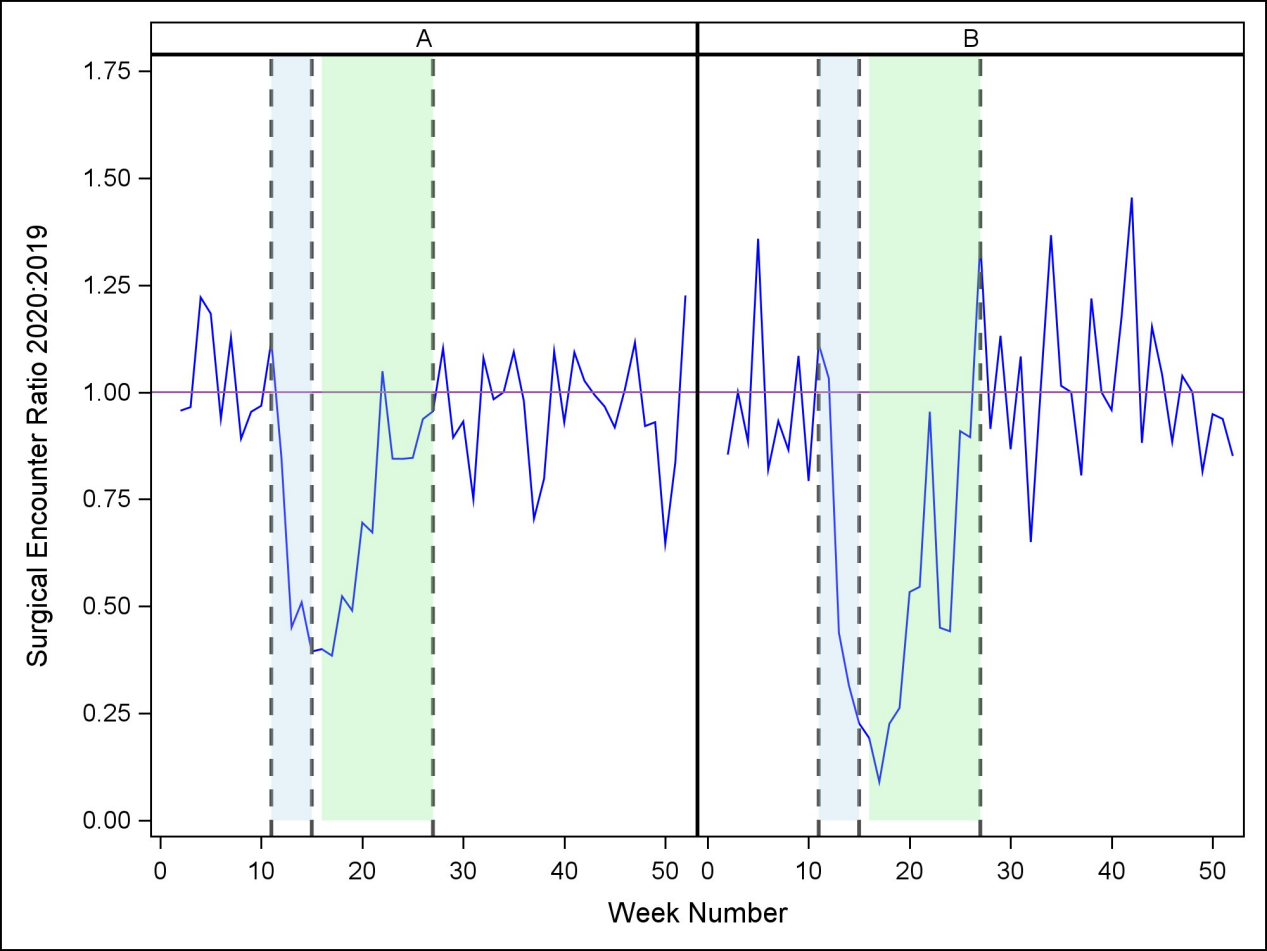
Weekly ratio (2020:2019) of surgical encounters, for each site. A = Allegheny Health; B = Johns Hopkins (Vertical reference lines denote periods beginning 3/8/20 (week 11), 4/5/20 (week 15), and 6/28/20 (week 27)).

Fig 1 illustrates the changes in surgical volume over these four periods, for each health system, comparing each week in 2020 to the corresponding week in 2019. Corresponding data are summarized in Table 1. During the pre-pandemic period (period 1), the ratio of surgical encounters in 2020 versus 2019 was close to 1.0, indicating that surgical volumes in 2020 were similar to 2019 during this period. Then, during period 2, surgical encounters exhibited a sharp negative trend (Allegheny Health: *p* < .0001, Johns Hopkins: *p* < .0001), to a nadir in period 3. During period 3, the mean surgical volume at Allegheny Health was 67% of the same period in 2019 and surgical volume was 48% of the same period in 2019 for Johns Hopkins. Also during period 3, surgical encounters exhibited a significant positive week-to-week trend (Allegheny Health: *p* < .0001; Johns Hopkins: *p* < .0001). Surgical volumes recovered in period 4, returning to levels that were not statistically different from period 1 (Allegheny Health 2020:2019 mean period 4 ratio of 0.96, *p* = 0.80, Johns Hopkins 2020:2019 mean period 4 ratio of 1.02, *p* = 0.88). Overall, 2020 surgical volume was 87% of 2019 volume at Allegheny Health (*p* = .0005) and 85% of 2019 volume at Johns Hopkins (*p* = .003).

**Table 1:** Total surgical encounters (for Allegheny Health (AH) and Johns Hopkins (JH), combined).

Time Period^§^	Total encounters		Ratio of mean encounters 2020:2019 (95% CI)
	2019	2020	
**1**	1965	1944	AH: 1.02 (0.9–1.14), JH: 0.95 (0.8–1.11)
**2**	845	615	AH: 0.73 (0.55–0.91), JH: 0.72 (0.49–0.96)
**3**	2597	1566	AH: 0.67 (0.57–0.78), JH: 0.48 (0.34–0.61)
**4**	5477	5305	AH: 0.96 (0.89–1.03), JH: 1.02 (0.93–1.11)

^**§**^ For 2020, period 1 was January 5 to March 7; period 2 was March 8 through April 4; period 3 was April 5 through June 27; period 4 was June 28 through December 27.

**Fig 2:**
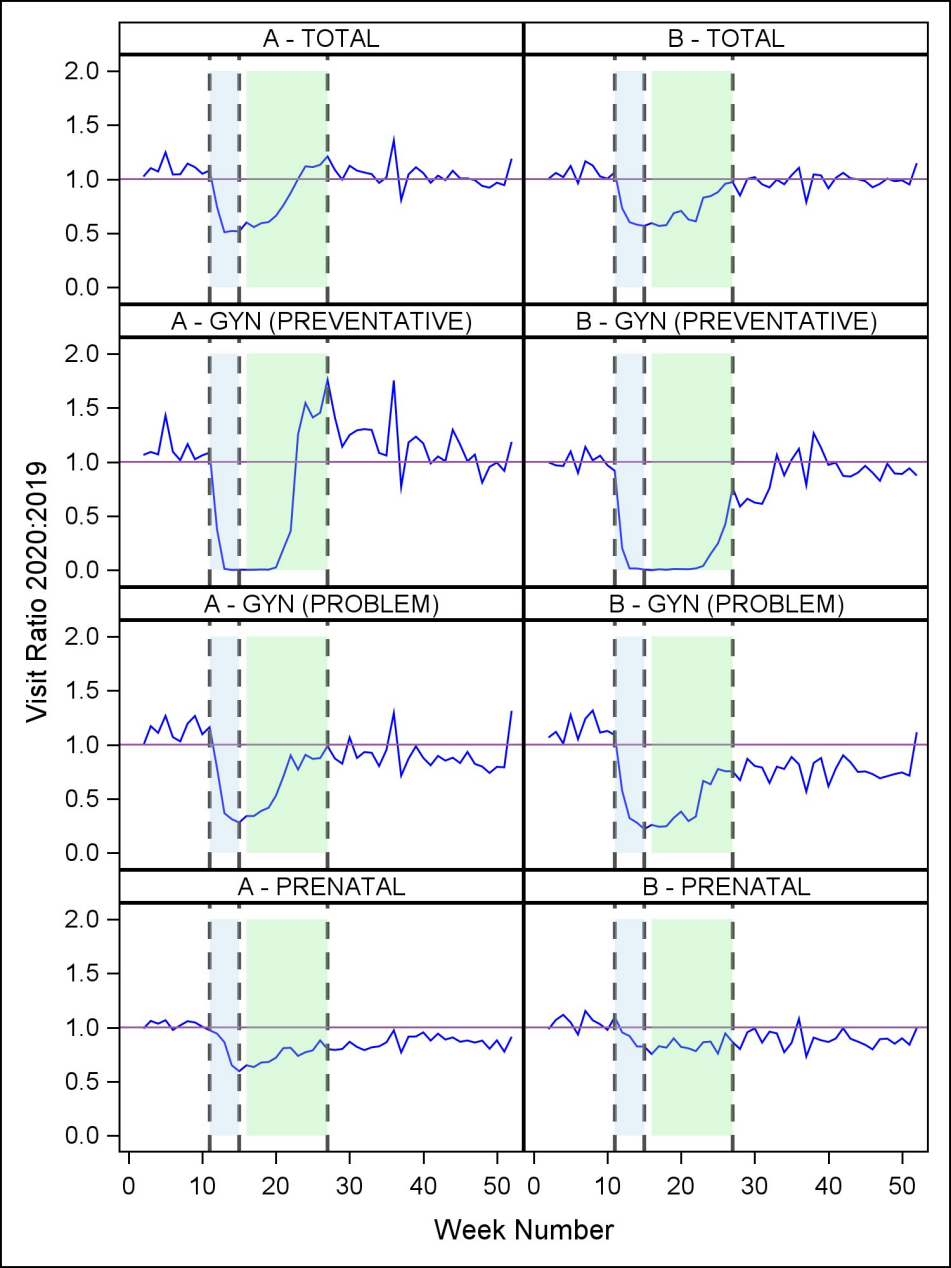
Weekly ratio (2020:2019) of ambulatory visits, by visit type, for each site. A = Allegheny Health; B = Johns Hopkins. The four panels illustrate the trends for all ambulatory visits as well as three visit subtypes: gynecologic (annual) preventative care, gynecologic problem-focused care, and prenatal care. (Vertical reference lines denote periods beginning 3/8/20 (week 11), 4/5/20 (week 15), and 6/28/20 (week 27)).

Similarly, there were fewer ambulatory in-person visits in 2020 versus 2019, although significance differed by site. 2020 volume was 96% of 2019 volume at Allegheny Health (*p* = .22) and 91% at Johns Hopkins (*p* = .0006). Fig 2 describes temporal changes in ambulatory visit volume for 2020 compared to 2019, with corresponding data in Table 2. The four panels illustrate the trends for all ambulatory visits as well as three visit subtypes: gynecologic (annual) preventative care, gynecologic problem-focused care, and prenatal care. Considering all ambulatory visits, the pattern was similar to that observed for surgical encounters. Specifically, in the pre-pandemic period, ambulatory encounters were similar in 2019 and 2020 (ratio approximates 1.0) with no significant week-to-week trend at either site (Allegheny Health: *p* = 0.92, Johns Hopkins: *p* = 0.78). In period 2, ambulatory visit volumes at both sites exhibited a steep negative trend (Allegheny Health: *p* < .0001, Johns Hopkins: *p* < .0001) followed by a significant positive trend in period 3 (Allegheny Health: *p* < .0001, Johns Hopkins: *p* < .0001). The mean ratios of ambulatory volumes during period 4 were statistically similar to pre-pandemic levels (Allegheny Health 2020:2019 mean period 4 ratio of 1.04, *p* = 0.81, Johns Hopkins 2020:2019 mean period 4 ratio of 0.98, *p* = 0.34).

**Table 2:** Total ambulatory visits (for Allegheny Health (AH) and Johns Hopkins (JH), combined).

Time Period^§^	Total visits		Ratio of mean visits 2020:2019 (95% CI)
	2019	2020	
**1**	60,689	65,406	AH: 1.09 (0.99–1.2), JH: 1.05 (0.98–1.12)
**2**	27,489	19,901	AH: 0.71 (0.55–0.87), JH: 0.74 (0.64–0.85)
**3**	80,528	61,385	AH: 0.79 (0.7–0.89), JH: 0.7 (0.64–0.76)
**4**	175,290	177,858	AH: 1.04 (0.98–1.1), JH: 0.98 (0.94–1.03)

^**§**^ For 2020, period 1 was January 5 to March 7; period 2 was March 8 through April 4; period 3 was April 5 through June 27; period 4 was June 28 through December 27.

Visits for gynecologic problem-focused care and prenatal care did not fully recover to 2019 levels in period 4. Specifically, in period 4, mean gynecologic problem visits were at 90% of 2019 levels at Allegheny Health, which was significantly lower than pre-pandemic levels (*p* = 0.02). Similarly, gynecologic problem visits at Johns Hopkins were only 77% of 2019 volumes at Johns Hopkins, which was also significantly lower than pre-pandemic levels (*p* < 0.0001). In period 4, 2020 prenatal visits were at 86% of 2019 volumes at Allegheny Health and lower than pre-pandemic levels (*p* < 0.0001) and at 89% of 2019 volumes at Johns Hopkins, also lower than pre-pandemic levels (*p* < 0.0001).

**Fig 3:**
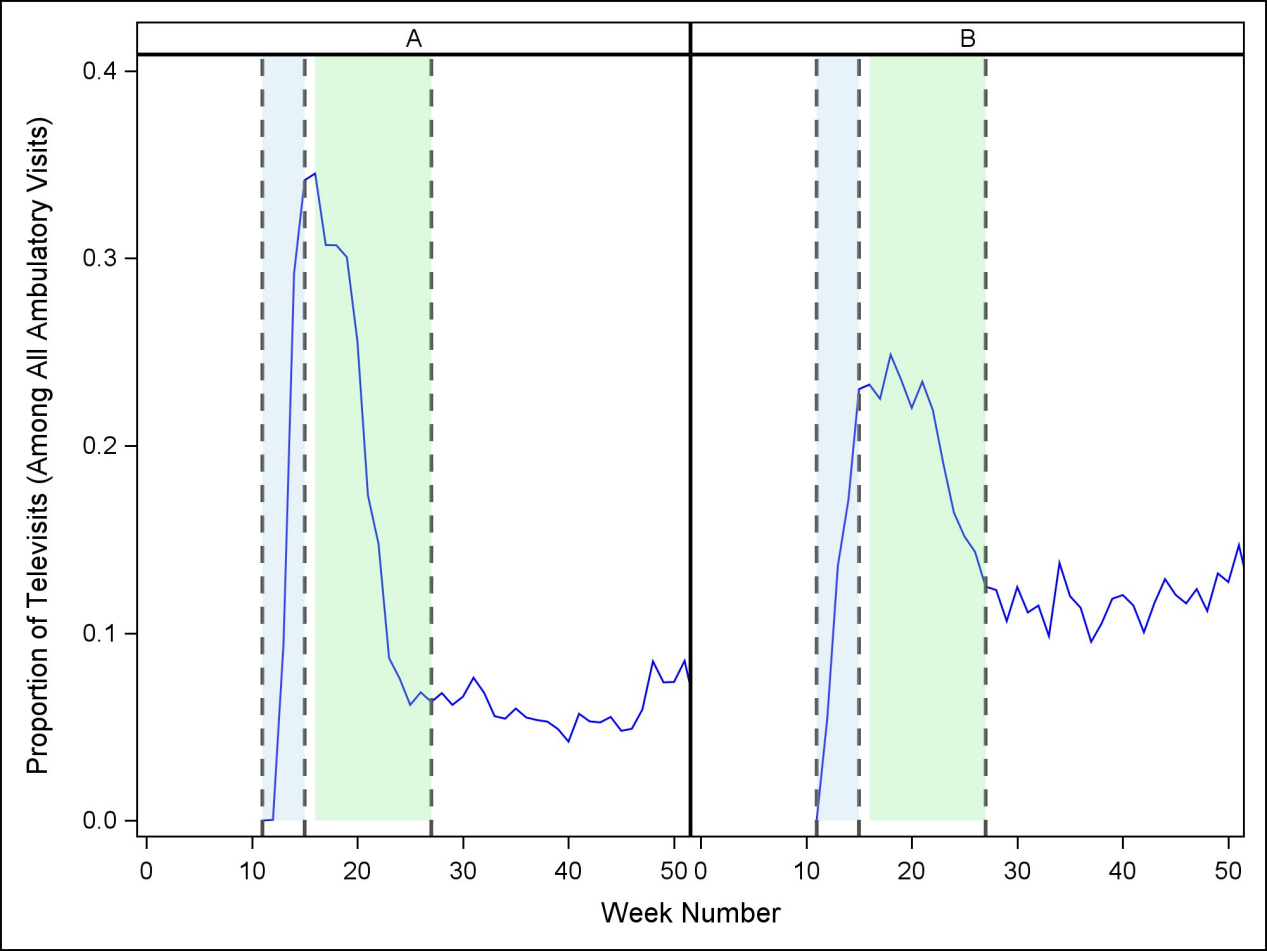
Proportion of 2020 ambulatory visits conducted as televisits, by week, for each site. A = Allegheny Health; B = Johns Hopkins (Vertical reference lines denote periods beginning 3/8/20 (week 11), 4/5/20 (week 15), and 6/28/20 (week 27)).

Fig 3 illustrates the proportion of visits conducted as televisits. During 2019 and the pre-pandemic period of 2020, televisits were negligible at both institutions. With the transition to period 2, the mean proportion of televisits rose sharply. By period 3, televisits represented approximately 21% of all visits for both sites, a significant increase (*p* < .0001 for both sites). In period 4, televisit use remained higher than prior to the pandemic: the proportion of televisits was 6% of all visits for Allegheny Health and 12% of all visits for Johns Hopkins. Televisit use was stable over time during period 4 but varied substantially among 5 subspecialties (Table 3). The utilization of televisits was highest for the Reproductive Endocrinology and Infertility group at Allegheny Health (89% of all visits) and lowest at both health systems for Female Pelvic Medicine and Reconstructive Surgery (less than 2% at Allegheny Health and approximately 5% at Johns Hopkins).

**Table 3:** Proportion of visits conducted as televisits, by subspecialty and by site (Allegheny Health versus Johns Hopkins) during period 4 (July—December 2020).

	Allegheny Health	Johns Hopkins
Reproductive Endocrine Infertility	89.5%	25.8%
Maternal Fetal Medicine	28.1%	18.7%
Family Planning and Contraception	14.0%	11.6%
Gynecologic Oncology	3.2%	10.4%
Female Pelvic Medicine and Reconstructive Surgery	1.7%	5.3%
**Gynecology and Obstetrics, overall**	**6.1%**	**11.8%**

**Fig 4:**
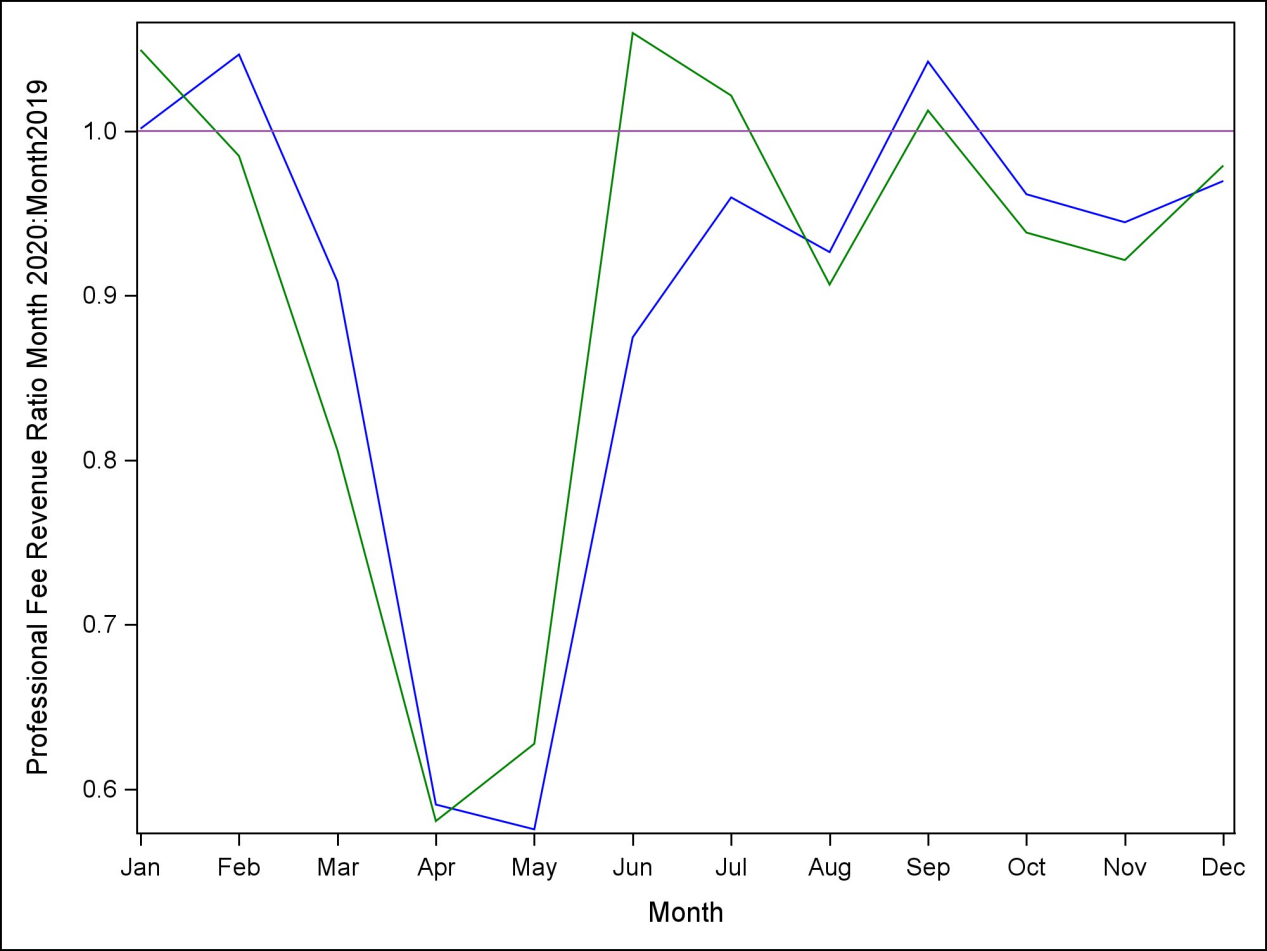
Ratio (2020:2019) of professional fee revenue, by month, for reach site. Blue line = Allegheny Health; Green line = Johns Hopkins.

Finally, Fig 4 illustrates the ratios of total monthly professional fee revenues in 2020 versus 2019. While revenues recovered in period 4, the total revenue for 2020 was only 91% of 2019 revenue for Allegheny Health and was only 90% of 2019 revenue for Johns Hopkins.

## Discussion

We observed a steep drop in gynecologic surgeries and ambulatory obstetrical and gynecologic visits at two large health systems during the COVID-19 pandemic. The reduction in surgical volume was especially pronounced from April 5 to June 27, 2020 (during period 3). This period coincided with the most restrictive institutional and regional policies, prohibiting or sharply restricting elective surgeries during this phase of the pandemic. Although we observed an increased volume of surgery in the second half of the year, surgical case load for 2020 did not recover to pre-pandemic levels. Similarly, ambulatory care declined substantially early in the pandemic and volumes of some types of ambulatory care did not recover to 2019 levels. This suggests that care was not simply delayed until after institutional restrictions had been relaxed in the latter half of 2020. At our two institutions, this led to a 9% decrease in professional fee revenue for all gynecological and obstetrical care. The financial impact of such losses may be substantial, as many health systems function on a tight 6–8% operating margin [19], and a 9% decrease in revenue is unlikely to be sustainable.

Other studies have documented a significant drop in a variety of primary and specialty health services provided in the first months of the 2020 pandemic. For example, data for insurance claims for over 6 million US adults [11] showed sharp declines in medical services during March and April of 2020, manifested as a reduction of almost 1500 ambulatory visits per 10,000 persons. Data from Kaiser Permanente in Southern California suggested an 80% reduction in ambulatory visits during this time period [20]. A similar reduction was noted by Chatterji and Li [21]. Reductions in surgical volumes have also been described, including for orthopedic surgery [11, 14], cataract surgery [11, 15], and cardiac surgery [13]. The data from the present study provides the ob/gyn specific perspective, including changes to gynecological surgery volumes and to ambulatory care in both obstetrics and gynecology. Due to the large variety of surgical procedure types performed at both institutions, we did not consider changes in individual gynecologic procedures or surgical indications. Gynecologic surgical practices during this public health crisis were likely influenced by published professional society guidelines for modifying surgical practice during the COVID-19 pandemic [22–24].

A critical question is whether the reduced utilization of obstetrical and gynecologic care during the early phase of the COVID-19 pandemic has had negative health consequences. Our study does not allow us to address the health impact of deferred or delayed care. For example, we observed a dramatic decrease in annual/ preventative gynecological visits during the worst of the pandemic. Others have demonstrated a reduction in other types of preventative care, including mammography [11, 25], screening for sexually transmitted infections [26], and cervical cytologic screening [21]. Concerns about the potential negative health outcomes associated with deferred or delayed care have been raised in the cancer, gynecologic and obstetric literature. Deferred or delayed care has been associated with more advanced breast and cervical cancers at initial diagnosis [27]. Modeling the effects of COVID-19 on breast and colon cancer screening and diagnosis over the next decade suggests an increase of 1% in cancer related deaths [28]. Furthermore, while a reduction in sexually transmitted infections may initially result from sexual distancing during the pandemic, reduced access to testing and treatment is expected to result in a rebound in incidence of sexually transmitted infections [29]. The reductions in care documented by our study raise important questions about the potential long-term health impact of deferred or delayed care.

An increased utilization of telemedicine, such as we observed in both health systems, could theoretically mitigate any negative impact of delayed care. Previous studies of telehealth interventions have demonstrated improvement in obstetric and gynecologic outcomes with telehealth, including regarding tobacco use, breastfeeding, medical abortion access, continuation of oral and injectable contraception, and need for high-risk obstetrical visits [30]. However, we found that telehealth adoption varied considerably among ob/gyn subspecialists. Also, these interventions may not be accessible to vulnerable populations [31], and thus the disproportionate impact on such populations must be considered.

The strengths of this study include robust data from two large health systems in two different states in the eastern United States. Interestingly, temporal patterns were quite similar for both institutions although the magnitude differed somewhat. However, these data may not be comparable to smaller U.S. health systems or those providing care in rural America.

A weakness of this study is that we did not examine how care may have been differentially impacted across various complaints or diagnoses. For example, with respect to gynecologic ambulatory care, we acknowledge that some “annual/ preventative care” visits likely included care for gynecologic problems or complaints. Also, we did not consider changes to obstetrical delivery volumes. The potential for COVID-19 to cause severe maternal morbidity and mortality has been recognized [32, 33]. We did not consider delivery volume during the pandemic, as we hypothesized these volumes would be more directly influenced by long-term trends in birth rates, rather than by temporal changes related to COVID-19. Lastly, due the retrospective nature of this study, we were unable to evaluate patient satisfaction with access to care and telehealth opportunities.

Our data clearly show a steep decrease in all gynecological and obstetrical care during the early pandemic. As the pandemic continues, it will be critical that ob/gyn clinicians develop new strategies to maintain or improve access to care while also maintaining safe processes that limit the spread of infections to patients and health care workers.

## Supporting information

S1 Table Current Procedural Terminology (CPT) codes used to identify gynecologic surgical procedures. (DOCX)
