# Supplementary material for: Impact of COVID-19 on gynecologic and obstetrical services at two large health systems
Source: PLoS One. 2022 Jun 16;17(6):e0269852. doi: 10.1371/journal.pone.0269852 (PMC9202837; doi:10.1371/journal.pone.0269852)
Supplement: S1 Table — (DOCX) [file pone.0269852.s001.docx]

**S1 Table: Current Procedural Terminology (CPT) codes used to identify gynecologic surgical procedures**

|  | CPT |
| --- | --- |
| Pelvic exenteration | 51597 |
| repair of bladder laceration | 51860 |
| MMK/Burch | 51840 |
| MMK/Burch complicated | 51841 |
| stamey suspension | 51845 |
| VVF closure abdominal | 51900 |
| Closure of vesicouterine fistula | 51920 |
| Closure of vesicouterine fistula with hysterectomy | 51925 |
| laparoscopic urethral suspension | 51990 |
| laparoscopic urethral suspension with sling | 51992 |
| cystoscopy | 52000 |
| cystoscopy with stent placement | 52005 |
| hydrodistension | 52260 |
| bladder biopsy without fulgeration | 52204 |
| bladder biopsy with fulgeration | 52224 |
| drainage of skene’s gland | 53060 |
| excision of urethral diverticula | 53230 |
| marsupialization of urethral diverticula | 53240 |
| excision of urethral polyp | 53260 |
| excision of urethral caruncle | 53265 |
| excision of skene’s gland | 53270 |
| excision of urethral prolapse | 53275 |
| urethroplasty | 53430 |
| urethrolysis | 53500 |
| repair of urethra | 53502 |
| Dilation of urethra | 53660 |
| Dilation of urethra subsequent | 53661 |
| dilation of urethra with general or spinal anesthesia | 53665 |
| transurethral radiofrequency | 53860 |
| placement of needles into pelvic organs for radioelement application | 55920 |
| Incision and drainage of vulvar abscess | 56405 |
| Incision and drainage of Bartholin’s | 56420 |
| marsupialization of Bartholin’s | 56440 |
| lysis of labial adhesions | 56441 |
| hymenotomy | 56442 |
| destruction of vulvar lesion (i.e. laser) | 56501 |
| destruction of vulvar lesion (i.e. laser) - extensive | 56515 |
| biopsy of vulva | 56605 |
| biopsy of vulva each separate lesion | 56606 |
| simple vulvectomy | 56620 |
| complete vulvectomy | 56625 |
| Radical vulvectomy- partial | 56630 |
| radical vulvectomy partial with unilateral nodes | 56631 |
| radical vulvectomy partial with bilateral nodes | 56632 |
| radical vulvectomy complete | 56633 |
| radical vulvectomy complete with unilateral nodes | 56634 |
| radical vulvectomy complete with bilateral nodes | 56637 |
| radical vulvectomy complete with inguinal and pelvic nodes | 56640 |
| partial hymenectomy | 56700 |
| excision of Bartholin’s | 56740 |
| plastic repair of introitus | 56800 |
| clitoroplasty for intersex state | 56805 |
| perineoplasty | 56810 |
| colposcopy of the vulva | 56820 |
| colposcopy of the vulva with biopsies | 56821 |
| colpotomy with exploration | 57000 |
| colpotomy with drainage of pelvic abscess | 57010 |
| colpocentesis | 57020 |
| Incision and drainage of vaginal hematoma obstetrical | 57022 |
| Incision and drainage of vaginal hematoma non obstetrical | 57023 |
| destruction of vaginal lesion (i.e. laser) | 57061 |
| destruction of vaginal lesion (i.e. laser) extensive | 57065 |
| biopsy of vaginal mucosa simple | 57100 |
| biopsy of vaginal mucosa extensive | 57105 |
| vaginectomy partial | 57106 |
| vaginectomy partial with removal of paravaginal tissue | 57107 |
| vaginectomy partial with removal of paravaginal tissue and nodes | 57109 |
| vaginectomy complete | 57110 |
| vaginectomy complete with paravaginal tissue removal | 57111 |
| vaginectomy complete with paravaginal tissue removal and nodes | 57112 |
| colpocleisis | 57120 |
| excision of vaginal septum | 57130 |
| excision of vaginal cyst | 57135 |
| irrigation of vagina or application of medication | 57150 |
| insertion of uterine tandem | 57155 |
| insertion of radiation apparatus | 57156 |
| introduction of hemostatic agent for nonobstetrical hemorrhage | 57180 |
| colporrhaphy of injury | 57200 |
| colpoperineorraphy of injury | 57210 |
| plastic operation of urethral sphincter (i.e. kelly) | 57220 |
| plastic repair of urethrocele | 57230 |
| anterior repair | 57240 |
| posterior repair | 57250 |
| anterior and posterior repair | 57260 |
| anterior and posterior repair with enterocele | 57265 |
| insertion of mesh vaginal approach | 57267 |
| repair of enterocele vaginal approach | 57268 |
| repair of enterocele abdominal approach | 57270 |
| colpopexy abdominal approach | 57280 |
| colpopexy vaginal approach (SSLF) | 57282 |
| colpopexy vaginal approach (USLS) | 57283 |
| paravaginal defect repair abd approach | 57284 |
| paravaginal defect repair vag approach | 57285 |
| removal of sling | 57287 |
| sling operation | 57288 |
| Pereyra procedure | 57289 |
| construction of artificial vaginal | 57291 |
| construction of artificial vagina with graft | 57292 |
| revision of vaginal graft vaginal approach | 57295 |
| revision of vaginal graft open approach | 57296 |
| RV fistula transvaginal or transanal approach | 57300 |
| RV fistula repair abd approach | 57305 |
| closure of RVF with colostomy | 57307 |
| closure of RVF transperineal approach | 57308 |
| closure of urethrovaginal fistula | 57310 |
| closure of urethrovaginal fistula with bulbocavernosus transplant | 57311 |
| closure of VVF vaginal approach | 57320 |
| closure of VVF transvesical and vaginal approach | 57330 |
| vaginoplasty for intersex state | 57335 |
| dilation of vaginal under anesthesia | 57400 |
| Examination under anesthesia | 57410 |
| removal of vaginal foreign body | 57415 |
| paravaginal defect repair laparoscopic approach | 57423 |
| laparoscopic colpopexy | 57425 |
| revision of prosthetic vaginal graft l/s | 57426 |
| colposcopy | 57452 |
| Colposcopy with biopsy | 57454 |
| Colposcopy with biopsy of cervix | 57455 |
| Colposcopy with ECC | 57456 |
| colposcopy with LEEP | 57460 |
| colposcopy with LEEP cone | 57461 |
| biopsy of cervix | 57500 |
| ECC | 57505 |
| cautery of cervix thermal | 57510 |
| cautery of cervix with cryo | 57511 |
| cautery of cervix with laser | 57513 |
| cone cold knife | 57520 |
| cone LEEP | 57522 |
| trachelectomy | 57530 |
| radical trachelectomy with nodes | 57531 |
| excision of cervical stump abd approach | 57540 |
| excision of cervical stump with prolapse repair | 57545 |
| excision of cervical stump vaginal approach | 57550 |
| excision of cervical stump vag approach with anterior and posterior repair | 57555 |
| excision of cervical stump with anterior and posterior repair and enterocele | 57556 |
| D&C of cervical stump | 57558 |
| cerclage non obstetrical | 57700 |
| trachelorrrahpy | 57720 |
| dilation of cervical canal | 57800 |
| D&C | 58120 |
| Myomectomy open | 58140 |
| myomectomy vaginal | 58145 |
| myomectomy 5+ abdominal | 58146 |
| total abdominal hysterectomy | 58150 |
| total abdominal hysterectomy with MMK | 58152 |
| supracervical hysterectomy | 58180 |
| total abdominal hysterectomy with nodes | 58200 |
| radical hysterectomy with nodes | 58210 |
| pelvic exenteration with total abdominal hysterectomy | 58240 |
| total vaginal hysterectomy <250g | 58260 |
| total vaginal hysterectomy <250g and adnexa | 58262 |
| total vaginal hysterectomy<250g with adnexa and enterocele | 58263 |
| total vaginal hysterectomy<250g with MMK | 58267 |
| total vaginal hysterectomy <250g with enterocele | 58270 |
| total vaginal hysterectomy with vaginectomy | 58275 |
| total vaginal hysterectomy with vaginectomy and enterocele | 58280 |
| total vaginal hysterectomy radical | 58285 |
| total vaginal hysterectomy >250 | 58290 |
| total vaginal hysterectomy >250 with adnexa | 58291 |
| total vaginal hysterectomy >250 with adnexa and enterocele | 58292 |
| total vaginal hysterectomy >250 with mmk | 58293 |
| total vaginal hysterectomy >250 with enterocele | 58294 |
| insertion of IUD | 58300 |
| removal of IUD | 58301 |
| introduction of saline or contrast for sonohyst | 58340 |
| transcervical introduction of fallopian tube cath | 58345 |
| insertion of heman capsule for brachytherapy | 58346 |
| chromotubation | 58350 |
| endometrial ablation | 58353 |
| endometrial cryoablation | 58356 |
| uterine suspension with shortening of round lig | 58400 |
| uterine suspension with presacral symphatectomy | 58410 |
| hysterorrhaphy for rupture uterine repair | 58250 |
| hysteroplasty repair of uterine anomaly | 58540 |
| laparoscopic ablation of fibroids | 58674 |
| laparoscopic supracervical hysterectomy <250 | 58541 |
| laparoscopic supracervical hysterectomy<250 with adnexa | 58542 |
| laparoscopic supracervical hysterectomy>250 | 58543 |
| laparoscopic supracervical hysterectomy>250 with adnexa | 58544 |
| laparoscopic myomectomy | 58545 |
| laparoscopic myomectomy 5+ | 58546 |
| laparoscopic rad hyst with nodes | 58548 |
| laparoscopic assisted vaginal hysterectomy <250 | 58550 |
| laparoscopic assisted vaginal hysterectomy <250 with adnexa | 58552 |
| laparoscopic assisted vaginal hysterectomy >250 | 58553 |
| laparoscopic assisted vaginal hysterectomy >250 with adnexa | 58554 |
| hysteroscopy | 58555 |
| hysteroscopy with biopsy | 58558 |
| hysteroscopy with lysis of adhesions | 58559 |
| hysteroscopy with resection of septum | 58560 |
| hysteroscopy with removal of fibroid | 58561 |
| hysteroscopy with removal of foreign body | 58562 |
| hysteroscopy with ablation | 58563 |
| hysteroscopy with fallopian tube perm implants | 58565 |
| total laparoscopic hysterectomy <250 | 58570 |
| total laparoscopic hysterectomy <250 with adnexa | 58571 |
| total laparoscopic hysterectomy>250 | 58572 |
| total laparoscopic hysterectomy>250 with adnexa | 58573 |
| total laparoscopic hysterectomy for malignancy with omentectomy | 58575 |
| ligation of fallopian tubes abd or vaginal | 58600 |
| ligation of fallopian tubes postpartum | 58605 |
| occlusion of fallopian tube with device | 58615 |
| laparoscopy with lysis of adhesions | 58660 |
| laparoscopy with removal of adnexa | 58661 |
| laparoscopy with fulguration of lesions | 58662 |
| laparoscopy with fulgeration of oviducts | 58670 |
| laparoscopy with fulgeration of oviducts and occlusion | 58671 |
| laparoscopic fimbrioplasty | 58672 |
| laparoscopic salpingostomy | 58673 |
| salpingectomy | 58700 |
| salpingo-oophorectomy | 58720 |
| lysis of adhesions | 58740 |
| tubotubal anastomosis | 58750 |
| tubouterine implantation | 58752 |
| fimbrioplasty | 58760 |
| salpingostomy | 58770 |
| drainage of ovarian cyst vag approach | 58800 |
| drainage of ovarian cyst abd approach | 58805 |
| drainage of ovarian abscess vag approach | 58820 |
| drainage of ovarian abscess abd approach | 58822 |
| transposition of ovary | 58825 |
| biopsy of ovary | 58900 |
| wedge resection of ovary | 58920 |
| ovarian cystectomy | 58925 |
| oophorectomy | 58940 |
| oophorectomy for malignancy | 58943 |
| resection of malignancy with BSO omentectomy | 58950 |
| resection of peritoneal malignancy with BSO, omentectomy and nodes | 58951 |
| resection of malignancy with BSO omentectomy, with radical dissection | 58952 |
| BSO with omentectomy, hysterectomy, radical dissection for debulking | 58953 |
| BSO, omentectomy, hysterectomy, debulking with nodes | 58954 |
| BSO with omentectomy, total abdominal hysterectomy for malignancy | 58956 |
| Tumor debulking of recurrent cancer | 58957 |
| Tumor debulking of recurrent cancer with nodes | 58958 |
| laparotomy for staging with nodes | 58960 |
| follicle puncture for oocyte retrieval | 58970 |
| embryo transfer | 58974 |
| intrafallopian tube transfer | 58976 |
| pudendal nerve block | 64430 |
| paracervical nerve block | 64435 |
| TAP block unilateral | 64486 |
| TAP block by continuous infusion | 64487 |
| TAP block bilateral | 64488 |
| TAP block bilateral by infusion | 64489 |
| Injection anesthetic agent superior hypogastric | 64517 |
| destruction by neurolytic agent superior hypogastric | 64681 |
| Lysis of adhesions >45 min | 44180 |
| sacroneuromodulation PNE | 64561 |
| sacroneuromodulation lead | 64581 |
| sacroneuromodulation IPG | 64590 |
| IPG removal | 64595 |
| lead removal | 64585 |
| martius flap | 20926 |
| sphincteroplasty | 46750 |
| trigger point injections | 20552 |
| trigger point injections >=3 sites | 20553 |
| insertion of port | 36561 |
| removal of port | 36590 |
| fluoro for port | 77001 |
| limited LAD staging | 38562 |
| laparoscopic retroperitoneal LN biopsy | 38570 |
| laparoscopic pelvic LAD | 38571 |
| laparoscopic pelvic LAD and peri aortic | 38572 |
| enterolysis | 44005 |
| enterectomy resection small intestine with anastomosis | 44120 |
| enteroenterostomy anastomosis small to large bowel | 44130 |
| mobilization of splenic flexure with partial colectomy | 44139 |
| colectomy partial with colostomy | 44141 |
| colectomy partial with end colostomy | 44143 |
| colectomy partial with coloproctostomy | 44145 |
| colectomy removal terminal ileum and ileocolostomy | 44160 |
| proctoscopy rigid | 45300 |
| suture small intestine single perf | 44602 |
| suture small intestine multiple perf | 44603 |
| appendectomy | 44955 |
| exploration retroperitoneal area | 49010 |
| ex <=5 cm intra-abdominal/retroperitoneal | 49203 |
| ex 5-10 cm intra-abdominal/retro | 49204 |
| exc >10 cm intraabd/retro | 49205 |
| omentectomy | 49255 |
| laparoscopy with biopsy | 49321 |
| placement of fiducial market | 49411 |
| insertion of intraperitoneal cannula | 49421 |
| removal of intraperitoneal cannula | 49422 |
| hernia repair | 49560 |
| ureterolysis | 50715 |
| Treatment of incomplete abortion | 59812 |
| Treatment of missed ab 1st trimester | 59820 |
| Treatment of missed ab 2nd trimester | 59821 |
| Treatment of septic abortion | 59830 |
| induced abortion by D&C | 59840 |
| Induced abortion by D&E | 59841 |
| induced abortion by intra-amniotic injections | 59850 |
| induced abortion by 1 or more intra amniotic injections | 59851 |
| induced abortion by 1_ intraamniotic injections with hysterotomy | 59852 |
| multifetal pregnancy reduction | 59866 |
| uterine evacuation and curettage for mole | 59870 |
